# Demagnetization Treatment of Remanent Composite Microspheres Studied by Alternating Current Susceptibility Measurements

**DOI:** 10.3390/ijms140918093

**Published:** 2013-09-04

**Authors:** Susanne van Berkum, Ben H. Erné

**Affiliations:** Van ’t Hoff Laboratory for Physical and Colloid Chemistry, Debye Institute for Nanomaterials Science, Utrecht University, Padualaan 8, 3584 CH Utrecht, The Netherlands; E-Mail: s.vanberkum@uu.nl

**Keywords:** magnetic microspheres, magnetic remanence, AC magnetic susceptibility, magnetic rheological probes, demagnetization treatment, Néel relaxation, Brownian relaxation

## Abstract

The magnetic remanence of silica microspheres with a low concentration of embedded cobalt ferrite nanoparticles is studied after demagnetization and remagnetization treatments. When the microspheres are dispersed in a liquid, alternating current (AC) magnetic susceptibility spectra reveal a constant characteristic frequency, corresponding to the rotational diffusion of the microparticles; this depends only on particle size and liquid viscosity, making the particles suitable as a rheological probe and indicating that interactions between the microspheres are weak. On the macroscopic scale, a sample with the dry microparticles is magnetically remanent after treatment in a saturating field, and after a demagnetization treatment, the remanence goes down to zero. The AC susceptibility of a liquid dispersion, however, characterizes the remanence on the scale of the individual microparticles, which does not become zero after demagnetization. The reason is that an individual microparticle contains only a relatively small number of magnetic units, so that even if they can be reoriented magnetically at random, the average vector sum of the nanoparticle dipoles is not negligible on the scale of the microparticle. In contrast, on the macroscopic scale, the demagnetization procedure randomizes the orientations of a macroscopic number of magnetic units, resulting in a remanent magnetization that is negligible compared to the saturation magnetization of the entire sample.

## 1. Introduction

Composite particles with embedded magnetic nanoparticles are becoming increasingly important in biomedicine. Well-known examples include particles used as contrast agents for MRI and particles with a chemically functionalized surface for the magnetic separation of specific biological molecules [1,2]. The magnetic properties of the composite particles result from the type of embedded nanoparticles. Magnetic nanoparticles usually have a single permanent magnetic domain and the magnetic anisotropy determines whether thermal reorientation of the dipole moment by the Néel mechanism is rapid or slow. Whether or not the composite microparticles have a permanent magnetic dipole moment depends on the orientations of the nanoparticle dipoles and the rate of Néel relaxation.

In most commercial applications, it is preferred to have magnetic microparticles without a permanent dipole moment. The advantage is that field-induced dipolar structures start to disappear as soon as the field is switched off. For magnetic separation applications, this means that the particles can be dispersed as single particles in their non-magnetized state and separated as large magnetic aggregates in their magnetized state. The disappearance rate of dipolar structures prepared in field is even at the basis of sensitive bio-assays that detect chemical interactions between the particles from a slowdown of the disaggregation rate [3]. Here, however, we choose to focus on microparticles with a permanent magnetic dipole moment, which have magnetic functional properties that are still relatively unexplored.

Microparticles with a permanent dipole moment have the advantage that their orientation can be detected magnetically. In principle, this makes them suitable as a rheological probe on the microparticle scale, which can also be used in environments that are not accessible optically. As long as the microparticles do not interact with each other, their rotational diffusion occurs on a time scale that is determined by the size of the particles and the viscosity of the medium:

(1) $$2\pi f_{B}=\omega_{B}=2D_{r}=\frac{k_{B}T}{4\pi \eta a_{h}^{3}}$$

where *f*_B_ is the Brownian relaxation frequency in Hz, ω_B_ is the radial frequency in s^−1^, *D*_r_ is the rotational diffusion coefficient, *k*_B_*T* is the thermal energy, η is the viscosity of the solvent, and *a*_h_ is the hydrodynamic radius of the microparticles. With magnetic particles, bio-assays have been developed that magnetically detect the slowdown of rotational diffusion that occurs when molecules adsorb to the particle surface, enlarging the hydrodynamic radius [4–8]. Moreover, cobalt ferrite nanoparticles have been used as rheological probes on the nanoscale [9] for instance to monitor the gelation of gelatin solutions [10] and to characterize the mechanical properties of ferrohydrogels [11].

**Figure 1:**
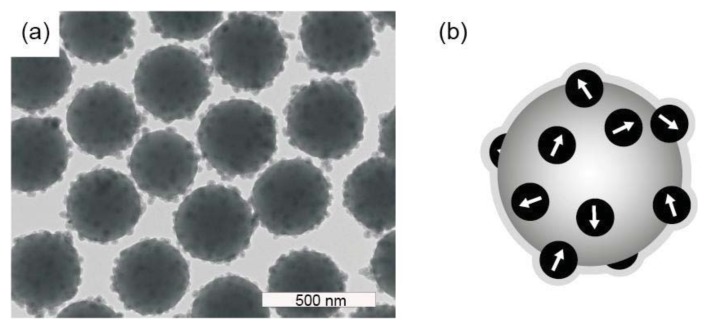
Silica microparticles with an embedded shell of cobalt ferrite nanoparticles: (**a**) TEM picture of the composite microspheres; and (**b**) schematic representation of the composite microspheres denoting the initial random orientation of the magnetic dipole moments. © IOP Publishing, 2007. Reproduced by permission of IOP Publishing. All rights reserved. doi:10.1088/0953-8984/19/3/036105 [12].

The remanent microparticles that we study here consist of monodisperse silica microparticles with a diameter of 380 nm and an embedded shell of cobalt ferrite nanoparticles with a diameter of 14 nm (Figure 1), particles developed by Claesson and Philipse [12]. An outer layer of silica weakens the dipolar interactions and prevents the formation of dipolar structures of the microparticles in an external magnetic field [13]. Their Brownian rotational motion was previously studied using frequency-dependent magnetic susceptibility measurements while the particles were dispersed in ethanol [14]. The weak interparticle interactions were shown to result in only a modest slowdown of rotational diffusion, up to concentrations where the microparticles self-assembled into colloidal crystals [14]. The weakness of the interparticle interactions, despite the permanent magnetic moment, in principle allows the microparticles to act as individual rheological probes.

Our objective here is to study the magnetic remanence of such microparticles in relation to their magnetic field history, by examining the effects of demagnetizing and remagnetizing the particles. Demagnetization procedures are well-known when it comes to bulk objects such as electromotors and magnetic data carriers [15]. Thermal demagnetization cannot be used in the case of colloidal dispersions of microparticles, because the solvent boils well before the Curie temperature of the magnetic material is reached and because the surface groups of the microparticles have a limited thermal stability. The particles might be demagnetized thermally in the dry state, but redispersing the particles in a solvent afterwards would be a challenge. To demagnetize our composite microparticles while in colloidal dispersion, we freeze the liquid and expose the sample to a magnetic field whose orientation alternates compared to that of the particles and whose magnitude is gradually decreased [15].

In the Results section, we first present our theoretical expectations on the basis of monodisperse embedded magnetic nanoparticles with fully blocked orientations of the magnetic dipole moments. This provides a framework to interpret the experimental results, which are complicated by polydispersity and Néel relaxation. Presentation of the experimental results starts with magnetization curves of the dry composite microparticles and of a dilute liquid dispersion of the separate nanoparticles, to determine the magnetic content per microparticle and the polydispersity of the nanoparticles. Next, magnetic remanence and its time dependence are characterized on the macroscopic scale, and the effect of carrying out a demagnetization procedure is demonstrated as well on the macroscopic scale. Finally, the AC magnetic susceptibility of liquid colloidal dispersions is measured as a function of frequency and amplitude, to characterize the magnetic remanence before and after demagnetization, now on the scale of the microparticles, and this is followed by a general discussion.

## 2. Results

### 2.1. Theory

The magnetic remanence of composite microspheres with embedded hard-magnetic nanoparticles depends on their magnetization history. This is discussed here theoretically, on the basis of embedded magnetic nanoparticles that are monodisperse and exhibit no Néel relaxation.

**Figure 2:**
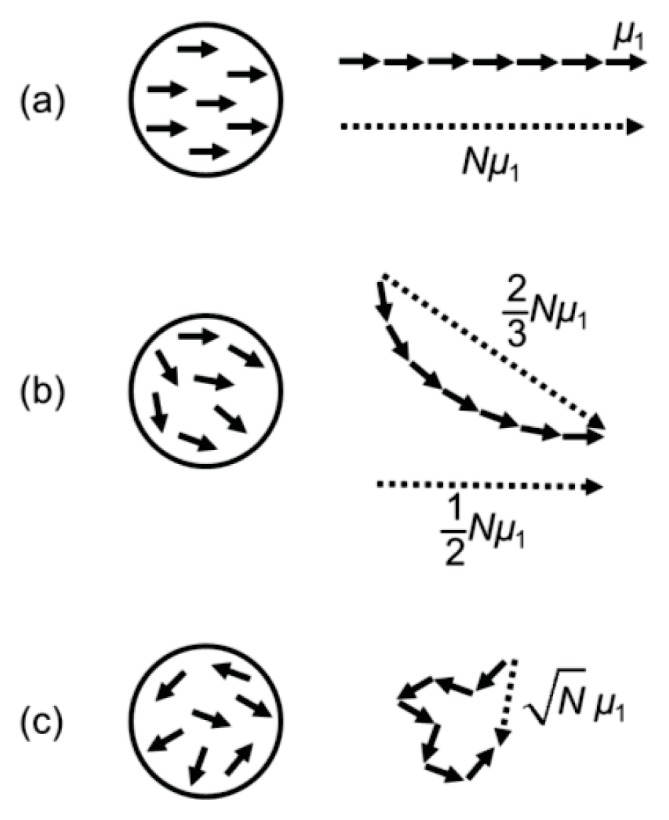
Schematic of the average net permanent dipole moment of remanent composite microparticles in three different magnetization states: (**a**) Saturation magnetization; (**b**) Maximum magnetization in zero field after saturation magnetization treatment; a distinction exists between the average magnetization in the direction of the previously applied field (½*N*μ_1_) and that in the direction of the net magnetic dipole of the microparticle (typically ⅔*N*μ_1_); (**c**) Minimum magnetization after randomization of the nanoparticle dipole orientations.

After treatment in a saturating magnetic field, the maximum remanence of composite microparticles is limited by the orientations and magnetic anisotropy energy of the embedded nanoparticles. In theory, full remanence could be attained if all the nanocrystals were identically oriented and if the anisotropy energy were sufficiently high compared to the thermal energy, so that it would prevent any orientational relaxation of the dipole moments in zero field. In that case, all the nanoparticle dipoles would point in the same direction along the same crystalline easy axis of magnetization. The microparticle dipole moment μ would then be equal to the number *N* of embedded nanoparticles times the dipole moment μ_1_ of a single nanoparticle: μ = *N*μ_1_ (Figure 2a).

A more realistic case is that the magnetic nanocrystals inside a microparticle are physically oriented at random. In zero field, the magnetization is then the vector sum of the nanoparticle dipoles with their various directions. Assuming that the easy axes of the nanoparticles are randomly oriented and that the dipoles have relaxed to the direction that is the closest to the previously applied field direction, the theoretical remanence in the field direction should on average be ½ of saturation magnetization (Figure 2b). Taking the magnetic field treatment to be along the *x*-axis, ½ is the average *x*-component when the *x*-components of randomly oriented unit vectors are suddenly all given a positive sign. Note that the direction of the net dipole of the microparticle in zero field does not have to be along the *x*-axis. When the frame of reference is taken to be the direction of the net dipole of each microparticle (Figure 2b), the average remanence typically tends to ⅔ (which we calculated by numerical simulation of the average vector sum of *N* randomly oriented unit vectors with a positive *x*-coordinate).

After a successful demagnetization treatment, the magnetization of the macroscopic sample as a whole should be completely negligible. On the scale of the individual microparticles, however, the situation is expected to be different. Even when all the nanoparticle dipoles inside one microparticle have random orientations, their vector sum is nonzero (Figure 2c). Mathematically, the sum of *N* randomly oriented unit vectors has a magnitude that is on average equal to the square root of *N*. In other words, the ensemble of nanoparticle dipole vectors describes a random walk. Therefore, the minimal remanence is nonzero. The same effect applies to the individual microparticles and to the entire macroscopic sample, but the scales are different. Relative to the total number *N* of nanoparticles present, the square root of *N* is much, much smaller in the case of the macroscopic sample (with its 10^12^ microparticles per 3 mL) than in the case of an individual microparticle (with only a few hundred nanoparticles). In this theory, the relative effect of demagnetization is inversely proportional to the square root of *N*.

### 2.2. Magnetic Content of the Composite Microspheres and Remanence on the Macroscopic Scale

**Figure 3:**
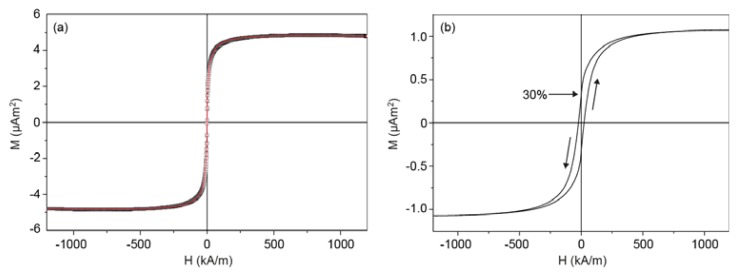
(**a**) Magnetization curve of cobalt ferrite nanoparticles in dispersion. The measured curve was fitted with Equation 4 to obtain the magnetic moment distribution of the nanoparticles; the measurements are in the open symbols and the fit is in red; (**b**) Magnetization curve of a macroscopic sample of dry composite microparticles (0.52 mg), characterizing the magnetic content and indicating a remanence of about 30% for the macroscopic sample. © IOP Publishing, 2007. Reproduced by permission of IOP Publishing. All rights reserved. doi:10.1088/0953-8984/19/3/036105 [12].

The magnetization *M* as a function of the applied magnetic field *H* was measured for cobalt ferrite nanoparticles in dilute dispersion and for dry composite microparticles made from such cobalt ferrite nanoparticles (Figure 3).

**Figure 4:**
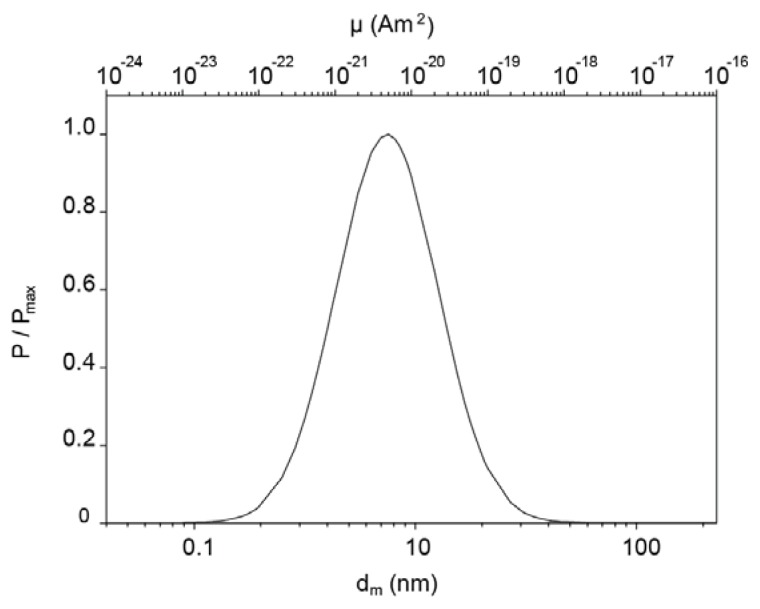
Distribution curves of the magnetic moment and the effective magnetic diameter of the cobalt ferrite nanoparticles, assuming lognormal distributions, calculated using Equations 6 and 7. *P* is scaled to the maximum of the distribution, *P*_max_.

The average dipole moment and polydispersity of the nanoparticles can be calculated from the magnetization curve of the nanoparticles in dilute dispersion. The measured curve does not show hysteresis, allowing a polydisperse fit on the basis of the Langevin equation and a lognormal distribution [16]. The Langevin equation describes how the magnetization *M* depends on the magnetic field:

(2) $$M=M_{s}\;L(\alpha )=M_{s}\;\left[\text{coth}(\alpha )-\frac{1}{\alpha }\right]$$

where *M*_s_ is the saturation magnetization,

(3) $$\alpha =\frac{\mu_{0}\mu H}{k_{B}T}$$

and μ_0_ is the magnetic permeability of vacuum, μ is the magnetic dipole moment of a nanoparticle, *H* is the magnetic field, and *k*_B_*T* is the thermal energy. For nanoparticles with a lognormal distribution and where interactions are negligible, the magnetization *M* at field *H* is given by

(4) $$M=M_{s}\frac{\int_{0}^{\infty }P(\mu )\;\mu \;L(\alpha (\mu ,H)\;d\mu }{\int_{0}^{\infty }P(\mu )\;\mu \;d\mu }$$

where *P*(*μ*) is the probability density function for μ. Equation 4 was used for a theoretical fit of the magnetization curve in Figure 3a to obtain the magnetic dipole moment of the individual nanoparticles. The magnetic dipole moment can be converted into an effective magnetic diameter *d*_m_ via

(5) $$\mu =\frac{\pi }{6}d_{m}^{3}\;m_{s}$$

where *m*_s_ is the material-dependent saturation magnetization per unit volume, 240 kA/m for cobalt ferrite, which was obtained from the magnetization curve in Figure 3a. This value for *m*_s_ is much lower than the bulk value of 425 kA/m reported in the literature, probably due to the presence of a non-magnetic iron oxide layer [17]. We assume that both the magnetic dipole moment and the magnetic diameter have a lognormal distribution:

(6) $$P(\mu )=\frac{1}{\sigma_{\mu }\mu \sqrt{2\pi }}\text{exp}\;\left[\frac{ln^{2}\left(\mu /\mu^{*}\right)}{2{\sigma_{\mu }}^{2}}\right]$$

(7) $$P(d_{m})=\frac{1}{\sigma_{\text{d}}d_{\text{m}}\sqrt{2\pi }}\text{exp}\;\left[\frac{ln^{2}\left(d_{m}/d_{m}^{*}\right)}{2{\sigma_{d}}^{2}}\right]$$

where *P* is the probability density function, μ^*^ and *d*_m_^*^ are respectively the dipole moment and magnetic diameter at the maximum of the distribution, and the width of the distribution is described by *σ*_μ_ = 3*σ*_d_. A fit of the data in Figure 3a yields a mean magnetic dipole moment μ = 1.61 ×·10^−19^ Am^2^ and σ_μ_ = 1.53, corresponding to *d*_m_*=* 11 nm and σ_d_ = 0.51. The positions of the maxima of the distributions are calculated using $\langle \mu \rangle =\mu^{*}\;exp[\sigma_{\mu }^{2}/2]$: μ_m_^*^ = 5.0·× 10^−2^ Am^2^ and *d*_m_^*^ = 9.7 nm. The lognormal distributions of the magnetic diameter and the probability density function of the magnetic moment are plotted in Figure 4.

The magnetic content of the microspheres was estimated from the magnetization curve of the dry microspheres (Figure 3b) and the bulk magnetization of cobalt ferrite: 42.1 mg of cobalt ferrite per gram of microspheres. Per silica microsphere, this corresponds to about 440 cobalt ferrite nanoparticles with a dipole moment of 1.61·× 10^−19^ Am^2^. The likelihood that the nanoparticles are present in small clusters due to magnetic interactions can be estimated from the distribution in Figure 4. The dimensionless dipolar contact interaction is given by

(8) $$\lambda =\frac{\mu_{0}\mu^{2}}{4\pi \;k_{\text{B}}T\;d^{3}}$$

where *d* is the particle diameter. About 13% of the nanoparticles is larger than 17 nm, corresponding to λ = 2, which is sufficient for nanoparticle aggregation [18,19]. This agrees with the presence of clusters in aqueous dispersions of cobalt ferrite nanoparticles of the type studied here as revealed by AC magnetic susceptibility measurements [20].

**Figure 5:**
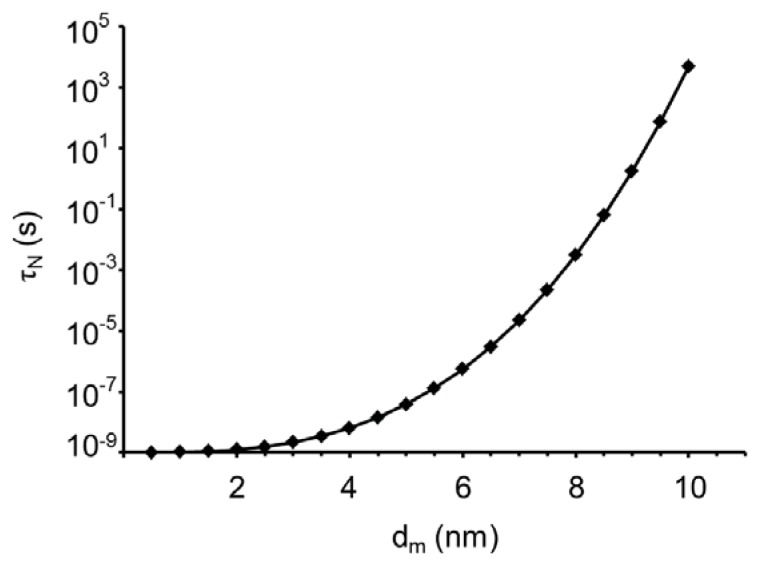
Characteristic Néel relaxation time τ_N_ as a function of the effective magnetic diameter *d*_m_ of cobalt ferrite nanoparticles (Equation 9).

Figure 3b indicates that the magnetic remanence of a dry sample of our microparticles is on the order of 30% after saturation magnetization treatment. This is significantly lower than the theoretically expected value of 50% (Figure 2b). The reason is that part of the embedded nanoparticles shows relatively rapid Néel relaxation of the dipolar orientation inside the nanoparticles. An indication for the Néel relaxation rate of the nanoparticles is given by

(9) $$\tau_{N}=\tau_{0}\;\text{exp}\;\left[\frac{KV_{\text{m}}}{k_{B}T}\right]$$

where τ_0_ is on the order of 10^−9^ s, *K* is the anisotropy constant, and *V*_m_ is the magnetic volume of the nanoparticles. The precise value of the anisotropy constant *K* for cobalt ferrite is not well known, with values of 120 kJ/m^3^ [21], 200 kJ/m^3^ [13,22], 180–300 kJ/m^3^ [23] and 3150 kJ/m^3^ [22,24] being quoted by different authors. For *K* = 120 kJ/m^3^, cobalt ferrite particles smaller than 9.5 nm exhibit Néel relaxation with τ_N_ < 100 s (see Figure 5), so that they do not contribute to the remanence on the time scale of our measurements of the magnetization curves. Comparing this to the magnetic size distribution in Figure 4 indicates that only about 60% of nanoparticles should contribute to the magnetic remanence in the magnetization curves of dry particles, in good agreement with 50% remanence expected without Néel relaxation and 30% remanence actually observed.

### 2.3. Demagnetization Treatment: Effect on the Macroscopic Scale

**Figure 6:**
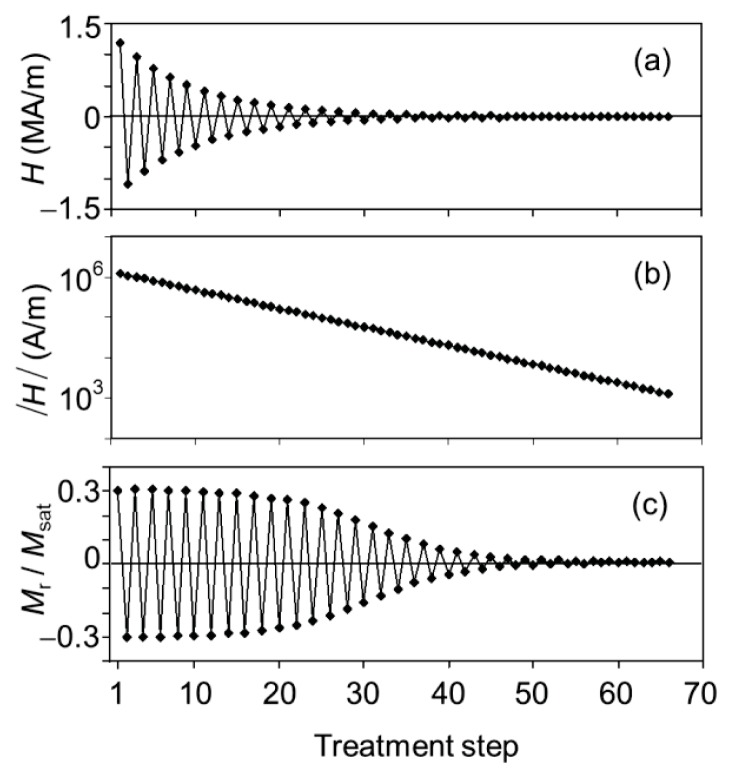
Demagnetization of a macroscopic sample of dry cobalt ferrite-embedded silica spheres: (**a**) Stepwise switching of the magnetic field *H* from positive to negative values of decreasing magnitude; (**b**) Absolute value of the applied field on a logarithmic scale; and (**c**) magnetic remanence *M*_r_ scaled to the saturation magnetization *M*_sat_.

Figure 6 illustrates how a demagnetization treatment affects our dry particles on the macroscopic scale. In each nanoparticle in zero field, the magnetic dipole prefers to be oriented along a crystallographically determined easy axis, and the nanocrystals are randomly oriented. The dipolar orientation can be reoriented using a magnetic field, but the strength of the required field depends on the orientations of the nanoparticles with respect to the field and on the volume of the nanoparticles. The nanoparticle dipoles are first all aligned in a strong field in a first direction, then a slightly weaker field is applied in the opposite direction, and so on (Figure 6a). When the field amplitude has dropped below about 400 kA/m (Figure 6b), most but not all the nanoparticle dipoles are reoriented at each field switch, and as the field continues to alternate and to weaken, fewer and fewer nanoparticle dipoles are reoriented by the alternating field (Figure 6c). The end effect is that the nanoparticle dipoles are left behind in random orientations, resulting in zero remanent magnetization.

**Figure 7:**
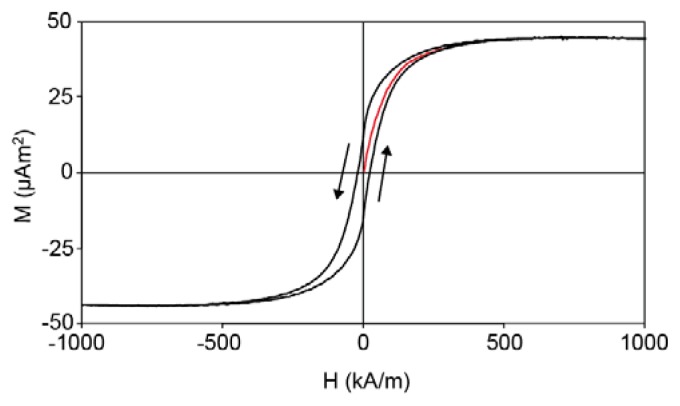
Magnetization curve of demagnetized composite microparticles. The initial loop (in red) begins at zero magnetization, indicating full demagnetization of the sample.

Figure 7 illustrates in another way that a sample of immobile microparticles could be demagnetized fully on the macroscopic scale. After demagnetization of a sample, the initial magnetization curve starts at the origin, that is, with zero magnetization in zero external field. The hysteresis loop indicates how strong the field must be to affect the orientations of the dipoles inside the immobilized nanoparticles. In the −400 kA/m to +400 kA/m range, the forward and backward scans are not exactly alike, in line with Figure 6 and the high coercivity of the cobalt ferrite particles [15].

### 2.4. AC Susceptibility Measurements

**Figure 8:**
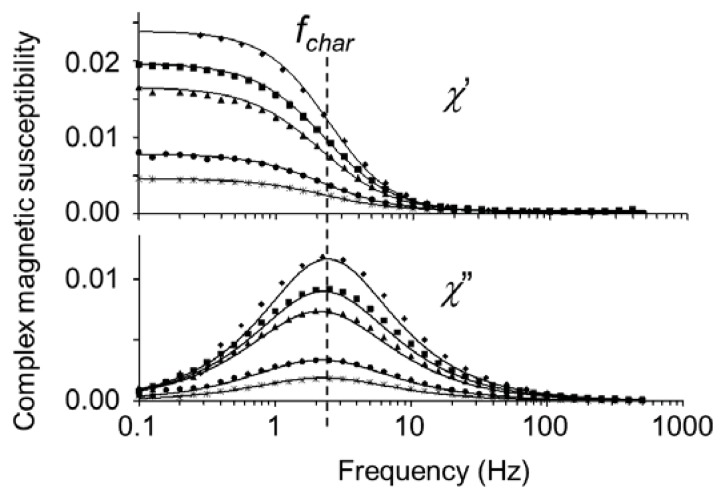
Frequency dependence of the real and imaginary components of the complex magnetic susceptibility χ = χ′ − jχ″ of cobalt ferrite-embedded silica spheres with a diameter *d* = 380 nm in ethanol at room temperature (170 A/m field amplitude). The same particles were magnetized to different extents after an initial demagnetization treatment; dependence of χ′ at 1 Hz on the magnetizing field is shown in Figure 9b. The relaxation at *f*_char_ = 2.5 Hz corresponds to the rotational diffusion rate of the microparticles.

To study the magnetic remanence on the scale of the microparticles, the AC magnetic susceptibility was measured as a function of frequency while the particles were dispersed in ethanol. The susceptibility χ = χ′ − jχ″ consists of an in-phase “real” component χ′ and an out-of-phase “imaginary” component χ″, both of which are plotted in Figure 8. Their dependence on the frequency *f* is given by

(10) $$\chi^{\prime }=\chi_{0}\frac{{f_{\text{char}}}^{2}}{{f_{\text{char}}}^{2}+f^{2}}$$

(11) $$\chi^{\prime \prime }=\chi_{0}\frac{f_{\text{char}}f}{{f_{\text{char}}}^{2}+f^{2}}$$

where χ_0_ is the low-frequency limit and *f*_char_ is the characteristic frequency [25]. Both components were numerically fitted jointly as a function of *f*, assuming a lognormal distribution of *f*_char_ [12,14]. The average characteristic frequency of 2.5 Hz is of the order expected for the Brownian rotation of particles with a diameter of 380 nm in ethanol (see Equation 1, with η = 1.074 mPa s, the viscosity of ethanol at 25 °C [12]). This indicates that magnetic relaxation of the sample requires rotation of the entire microparticles. In other words, the magnetic susceptibility is determined by the number of microparticles and their net permanent dipole moments, as opposed to being due to the Néel relaxation of the dipoles inside individual embedded nanoparticles [12]. From the polydispersity of *f*_char_, a polydispersity of about 15% was calculated for the hydrodynamic radius, in agreement with a polydispersity of 18% from electron microscopy [12]. The different spectra were obtained by magnetizing the same particles at different fields after an initial demagnetization treatment. The characteristic frequency is practically the same in all the spectra, indicating that the rotation remains that of single microparticles, as opposed to magnetic assemblies of particles, which would relax at much lower frequencies [26].

**Figure 9:**
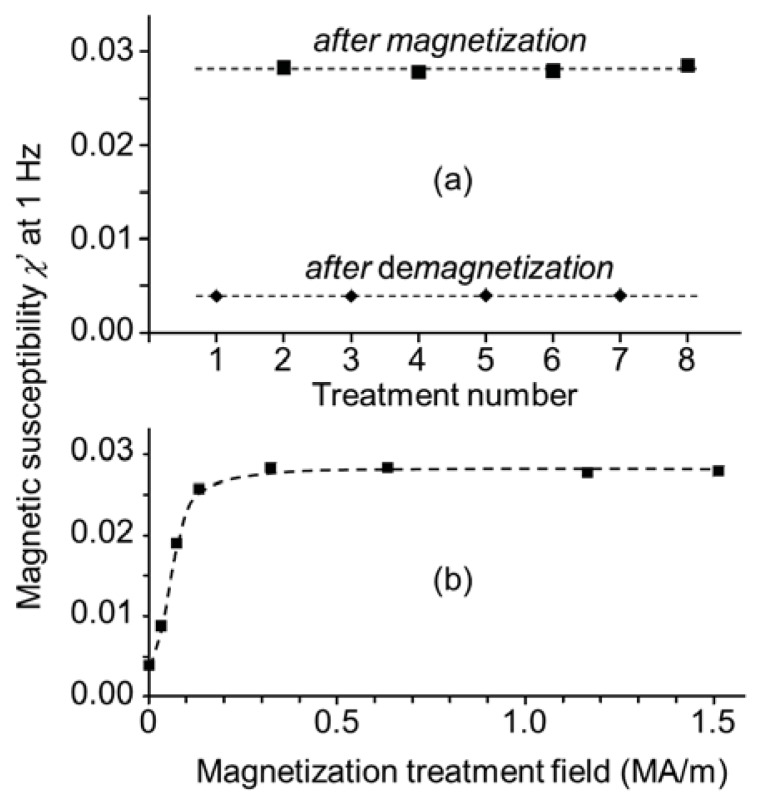
Real component χ′ of the magnetic susceptibility χ = χ′ − jχ″ at 1 Hz for the remanent microsphere dispersions in ethanol: (**a**) After alternating treatments to magnetize at 1.5 MA/m or to demagnetize; (**b**) After demagnetization and remagnetization as a function of the remagnetization field strength. The demagnetization and remagnetization treatments were at 77 K, whereas the susceptibility was measured at room temperature.

To demagnetize the magnetic microparticles in colloidal dispersion, we froze the solvent of the dispersion using liquid nitrogen and rotated the frozen dispersion with respect to a magnetic field of fixed orientation but decreasing magnitude (see Section 3.2). During the demagnetization treatment, the nanoparticles respond individually to the magnetic field whereas the microparticles are unable to physically rotate in the frozen solvent. Whether or not the nanoparticles are present in microparticles does not affect the response of the nanoparticles. The susceptibility at 1 Hz after consecutive demagnetization or remagnetization treatments is shown in Figure 9. The maximum susceptibility after magnetization treatment was a factor 7 higher than the minimum after demagnetization treatment, meaning that the dipole moment was higher by a factor of 2.7, since magnetic susceptibility scales with the square of the dipole moment. Intermediate values were obtained by magnetizing in fields lower than 400 kA/m, when the in-field magnetization does not yet saturate the sample at 77 K.

**Figure 10:**
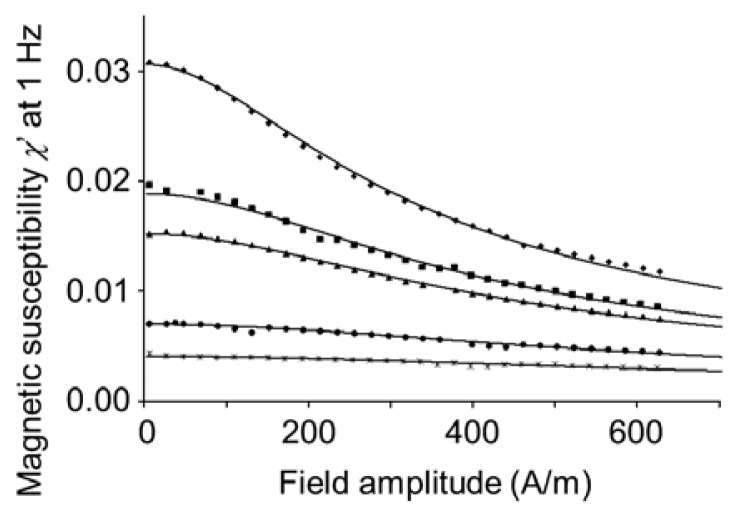
Field amplitude dependence of the real component χ′ of the magnetic susceptibility χ = χ′ − jχ″ at 1 Hz (same samples as in Figure 9). The fitted values of the average magnetic dipole moment per microparticle, from top to bottom, were 3.01, 2.41, 2.21, 1.64, and 1.32 in units of 10^−17^ Am^2^. This corresponds to 175, 138, 126, 96, and 75 times 1.6·× 10^−19^ Am^2^, the average dipole moment of the embedded nanoparticles.

In principle, the microparticle dipole moment μ can be calculated from the low-frequency limit of the magnetic susceptibility, χ_0_, and the number *N* of microparticles present per unit volume *V*, because

(12) $$\chi_{0}=\frac{N\mu_{0}\mu^{2}}{3k_{\text{B}}TV_{0}}$$

where μ_0_ = 4π·× 10^−7^ J A^−2^m^−1^. However, the dipole moment can be determined more reliably from the low-frequency limit of the magnetic susceptibility as a function of the amplitude *H* of the applied alternating magnetic field, since this does not require precise knowledge of the concentration *N*/*V* [12]:

(13) $$\frac{\chi_{0}(H)}{\chi_{0}(H\to 0)}=\frac{L(\alpha )}{\alpha /3}$$

where *L*(α) and α are given by Equations 2 and 3. Such data is presented in Figure 10. The observation that the susceptibility does not increase but only decreases at increasing field amplitude is direct evidence of a permanent rather than an induced dipole moment of the microparticles [12,27]. The fits and the calculated dipole moments assume that the average alignment of the microparticle dipoles in an external magnetic field is given by the Langevin function [12]. Assuming a lognormal distribution, the fitted polydispersity of the dipole moment was on the order of 30% after saturation magnetization treatment. This agrees with the 15% polydispersity in the microparticle radius and the fact that the nanoparticles are located in a spherical monolayer shell.

**Figure 11:**
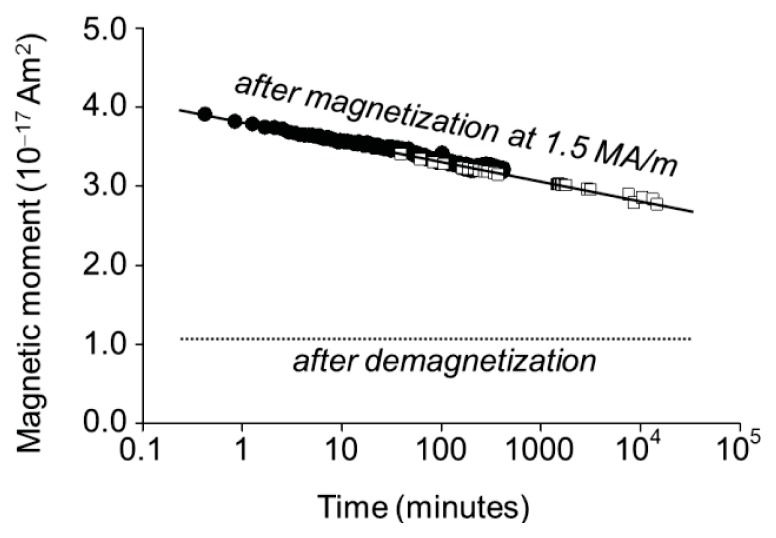
Slow decrease of the magnetic dipole moment of the microparticles after magnetization at 1.5 MA/m. The plot combines AC susceptibility data (calculated from χ_0_, open squares) and, at shorter times, rescaled remanent magnetization data acquired with the alternating gradient magnetometer on dry particles (filled circles).

The AC susceptibility measurements are relatively insensitive to nanoparticles with rapid Néel relaxation, since χ depends on the square of the dipole moment (Equation 12). The contribution due to Brownian rotation of a microparticle with a dipole moment μ *= N ×* μ_1_*is* proportional to *N^2^*, whereas the contribution of *N* nanoparticles with a dipole moment μ_1_ that respond individually by Néel relaxation is proportional to *N*. Nevertheless, the AC susceptibility measurements do show evidence of Néel relaxation, be it on time scales of minutes to weeks: a slow decrease of the remanent magnetization (Figure 11). The decrease in remanence with the logarithm of time is as expected for frozen ferrofluid spin glasses [28,29]. Two factors that affect the rate of decrease are the polydispersity in the nanoparticle dipole moments and the interactions between the dipoles.

## 3. General Discussion

The dipole moments of our composite microparticles can now be compared to the theory (Figure 2). From the saturation magnetization of the microparticles (Figure 3), it was concluded that a microparticle contains about 440 nanoparticles. After treatment in a saturating magnetic field, the dipole moment from AC susceptibility measurements was found to be 175 × μ^1^ (Figure 10), with μ^1^ = 1.6·× 10^−19^ Am^2^ being the average dipole moment of a single nanoparticle. This is a remanence of 40%, compared to 30% remanence indicated by the magnetization curve of dry particles (Figure 3). However, the remanence of 30% was measured in the direction of the previously applied field, whereas the remanence of 40% is the average per particle along the direction of magnetization of each microparticle in zero external field. From theory, the remanence in the direction of the net microparticle dipole (⅔*N ×* μ_1_) is higher than in the direction of the initially applied field (½*N ×* μ_1_) by 1/3, which agrees with our experimental data.

Theoretical agreement with the magnetic moment after demagnetization treatment is not as clear. The theoretical prediction is $\sqrt{N}\times \mu_{1}$, and probably the 40% of particles with rapid Néel relaxation should not be included in *N*. This leads to *N* = 264 and a predicted remanence of 21 × μ_1_, compared to 75 × μ_1_ from AC susceptibility measurements. A possible explanation for this discrepancy is the presence of clusters in which magnetic nanoparticles are in the head-to-tail configuration rather than in a flux closure configuration, which would suppress the remanence. In that way, the randomly oriented magnetic units that have to be taken into account have a larger dipole moment, for instance 10 × μ_1_, and there are fewer of such units, for instance *N*/10 = 26. This will then lead to a higher remanence of order $\sqrt{26}\times 10\times \mu_{1}=51\times \mu_{1}$.

The theoretical predictions clearly give an oversimplified picture, and experimental agreement is not quantitative. Nevertheless, two general predictions seem to be confirmed by our data: (1) the average remanence after treatment in a saturating field is higher along the direction of each microparticle dipole than in the direction of the initially applied field, and (2) a demagnetization treatment does not lead to the full demagnetization of the individual microparticles, even though the sample appears to fully lose its remanence on the macroscopic scale. From theory, the relative effect of demagnetization is inversely proportional to the square root of the number *N* of nanoparticles, where *N* is huge for macroscopic samples (here per 3 mL there are typically 10^12^ microparticles with each about 440 nanoparticles) and much smaller for a single microparticle (here about 440). In practice, the difference in magnetic remanence between the lowest and the highest magnetization states in zero field should increase as the number of nanoparticles embedded per microparticle increases, and it should decrease when the nanoparticles interact with each other magnetically.

The maximum extent to which remanent composite microspheres can be demagnetized may not only be of fundamental but also of practical interest. Remanent microparticles with stronger magnetic interactions than in our work can self-assemble into dipolar structures in zero field, in a similar way as has been observed with single-domain magnetic nanoparticles [18,19,30]. Since the microparticles can be magnetized or demagnetized by magnetic treatment, this can lead to the appearance or disappearance of zero-field dipolar structures, as was recently shown by Smoukov *et al.* [31]. In the low-magnetization state, the particles do not interact strongly with each other and can be dispersed in a liquid as single particles, resulting in a stable colloidal dispersion that is convenient to handle. In the high-magnetization state, dipolar structures grow that are likely to settle much more rapidly to the bottom of the dispersion.

## 4. Experimental Section

### 4.1. Magnetically Remanent Silica Microspheres

The experiments were performed on silica microspheres containing an embedded monolayer shell of cobalt ferrite nanoparticles. The synthesis of the particles is briefly summarized here and was described in detail by Claesson and Philipse [13]. Cobalt ferrite nanoparticles were prepared separately by aqueous coprecipitation [32], treated with HNO_3_ and Fe(NO_3_)_3_·9H_2_O solutions, and washed with water. In parallel, silica microparticles were prepared by the Stöber method [33], from tetraethyoxysilane in a mixture of ethanol, water, and ammonia. The silica particles were grafted with mercaptopropyl(trimethoxy)silane (MPTMS) to ensure that the subsequent adsorption of a monolayer of cobalt ferrite particles was irreversible. Finally, a shell of silica was added using the Stöber method, resulting in the particles characterized as “Sample 1” in [12]. They were surface-coated with 3-(trimethoxysilyl)propyl methacrylate for steric colloidal stabilization and dispersed in ethanol at a concentration of 3.0·× 10^17^ particles per m^3^ (0.86 vol.%).

### 4.2. Demagnetization of Liquid Dispersions

**Figure 12:**
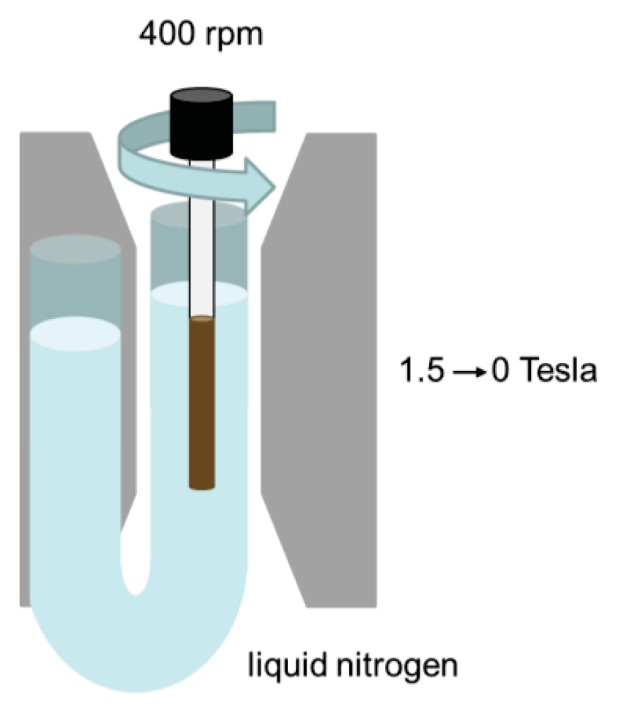
Schematic illustration of the setup used to demagnetize the microparticles while dispersed in ethanol.

Samples with 3 mL of colloidal dispersion in a glass sample tube with an internal diameter of 6 mm were demagnetized as follows (Figure 12).

The sample tubes were immersed in liquid nitrogen while being rotated at 400 rpm between the poles of a Bruker BE 25 V electromagnet (pole caps of 11 cm by 22 cm, 4 cm apart). The sample was held vertically by a rotating holder that is connected via a rubber transmission band to an electromotor situated 30 cm from the pole caps, where it is not disrupted by the magnetic field. The sample hung inside one end of a vertical U-tube prepared from flexible thermal insulating tubing that could be rapidly filled with liquid nitrogen by pouring it into the other end. The field was increased to 1.5 MA/m (1.9 Tesla) in 10 s at room temperature, 240 s were taken to add liquid nitrogen to freeze the sample, after which rotation was started and the field was linearly decreased to zero in 150 s. Remagnetization was also done cryogenically, without sample rotation. Caution should be taken when applying this procedure to aqueous samples, since in contrast to ethanol, water expands upon freezing.

### 4.3. Magnetic Measurements

Frequency-dependent measurements of the complex magnetic susceptibility were measured on 3 mL samples of the colloidal dispersion using a homebuilt setup [34]. Measurements were done at 295 K, from 0.1 to 500 Hz using a sinusoidal magnetic field of 170 A/m in amplitude, and at 1 Hz as a function of the magnetic field amplitude from 6 to 600 A/m.

Smaller scale experiments were conducted on dry samples (8 mg of microparticles) using a Micromag 2900 alternating gradient magnetometer (Princeton Measurements Corporation, Princeton, NJ, USA) at room temperature. Magnetization curves were obtained at room temperature at a scanning rate of 25 kA/m per s. Also with this apparatus, samples can be demagnetized by an automatic procedure or manually, by setting different positive or negative field values in any desired sequence. The equipment was also used to measure how fast the remanent magnetization of the particles decreased on time scales of 1 to 1000 min. Time-dependent remanence measurements on time scales of 30 min to several weeks were measured using the homebuilt complex magnetic susceptibility meter.

## 5. Conclusions

The remanence of our microparticles with embedded cobalt ferrite nanoparticles does not become zero after a demagnetization treatment. The macroscopic sample appears fully demagnetized, but on the scale of individual microparticles, the number of randomly oriented magnetic clusters of nanoparticles is so small that the vector sum of their magnetic dipoles is not negligible. This illustrates a key difference between magnetization curves and AC susceptibility as a way to characterize the magnetic remanence of microspheres. Whereas magnetization curves give an average over all magnetic particle dipoles in the macroscopic sample, magnetic susceptibility spectra of liquid dispersions give an average magnetic dipole moment on the scale of the microspheres.
